# Simple understanding of quantum weak values

**DOI:** 10.1038/srep20286

**Published:** 2016-02-03

**Authors:** Lupei Qin, Wei Feng, Xin-Qi Li

**Affiliations:** 1 Center for Advanced Quantum Studies and Department of Physics, Beijing Normal University, Beijing 100875, China; 2 Department of Physics, Tianjin University, Tianjin 300072, China

## Abstract

In this work we revisit the important and controversial concept of quantum weak values, aiming to provide a simplified understanding to its associated physics and the origin of *anomaly*. Taking the Stern-Gerlach setup as a working system, we base our analysis on an exact treatment in terms of quantum Bayesian approach. We also make particular connection with a very recent work, where the anomaly of the weak values was claimed from the pure statistics in association with “disturbance” and “post-selection”, rather than the unique quantum nature. Our analysis resolves the related controversies through a clear and quantitative way.

The concept of weak values (WVs), introduced by Aharonov, Albert and Vaidman (AAV) nearly 30 years ago^1^,^2^, has caused continuous interests and controversies^3^,^4^,^5^,^6^,^7^,^8^,^9^,^10^,^11^,^12^,^13^. A large number of references can be found, for instance, in the recent review articles^13^,^14^,^15^. The simple reason for causing both interests and controversies might be seen from the unusual form of the AAV WV:


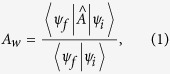


where 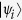 and 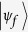 are the pre- and post-selected (PPS) states of 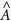. The most surprising prediction of this formula is that it can drastically exceed the range of eigenvalues of the observable 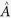, violating thus our common knowledge.

The AAV WV formula itself, i.e., Eq. (1), was obtained based on quantum mechanics. Therefore, unlike emphasized in refs 10,13, it seems not so surprising that there is no analogous formula based on classical principles which can display the *functional* feature of Eq. (1). However, as properly pointed out in refs 10,13, the appearance of *anomalous* WV based on Eq. (1) (exceeding the range of the eigenvalues of 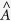) is indeed originated from the *quantum interference*^10^,^13^. In ref. 13, the anomalous WV is also related with *negative* quasiprobabilities, which highlights further the quantum nature of the AAV WV.

Moreover, in ref. 10, the quantum nature of the AAV WV has been elaborated further as follows. Consider two coupled systems (or the degrees of freedom of a single system), say, “*A*”-plus-“*B*” with coupling Hamiltonian 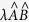. (In the WV studies, “*B*” is utilized as the meter for quantum measurements). The weak value *A*_*w*_ of 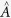 is defined by the PPS states of the system “*A*”, as given by Eq. (1). *A*_*w*_ plays the role of an effective parameter coupled to 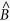 and results thus in a “pre-existing” *shift* in the wave function of the system “*B*”. This understanding has been highlighted in particular as^10^: “The weak value shifts exist if measured or not, so the weak value is not defined by the statistics of measurement outcomes. The statistical analysis (performed after the post-selection) can just reveal the pre-existing weak values.” This particular statement was mainly directed to the recent work by Ferrier and Combes^7^, in which (and in the later response article^8^) the *anomaly* of the WV was claimed from a reason of pure statistics associated with *disturbance* and *post-selection*, rather than the unique quantum nature.

Actually, the work by Ferrier and Combes^7^ is just the latest of a series of works in the past years on classical analogues of the WVs and associated paradoxes^16^ followed by appropriate clarifications^17^,^18^. Discussions on the classical- *versus*-quantum issues were also put forward in different manners, from aspects such as violation of the Leggett-Garg inequality^19^, negative quasiprobability^13^, contextual values (contextuality)^20^,^21^,^22^,^23^, and even the nature of time^24^.

In this work we present a simple, explicit, and quite straightforward way to understand how the AAV WV appears as or enters the PPS average of the measurement outcomes, and how the *anomaly* is caused. For the whole problem, two points are essential: one is the post-selection conditioned average; the other is the superposition principle of quantum mechanics. We will base our analysis on an exact treatment in terms of quantum Bayesian approach by taking the Stern-Gerlach setup as a working system. The reason of using Bayesian approach is twofold: (i) it enables to easily obtain the *exact* result (for arbitrary measurement strength) which will serve as the unified starting point for the whole analysis in this work; and (ii) it allows to clarify that any classical model under correct treatment cannot result in anomalous WVs. We notice that this second point does not arrive to full consensus in literature. We believe that the present work can, in a transparent and quantitative way, resolve the recent controversies^7^,^8^,^9^,^10^,^11^,^12^,^13^.

## Results

### AAV’s Weak Values

**Figure 1:**
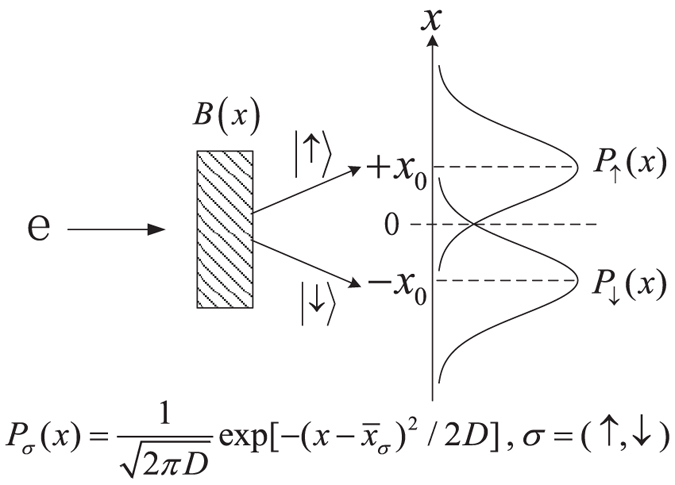
Schematic plot of the Stern-Gerlach setup utilized for studies of weak measurement and quantum weak values.

For the sake of completeness let us briefly review the AAV’s treatment of weak values, by taking the Stern-Gerlach setup as a specific working system, as schematically shown in Fig. 1. In this setup the electron’s trajectory is deflected when it passing through inhomogeneous magnetic field. Corresponding to “
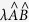
”, now the interaction Hamiltonian between the “system” and the “meter” reads 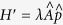. That is, the spin degree of freedom of the electron is the system and the spatial ones (momentum and coordinate) are the meter. In this work we use 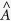 for the spin operator. Let us consider the system and meter starting the evolution with 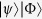 where 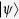 and 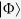 are, respectively, the system and meter states. For the meter state, i.e., the transverse wavefunction (wavepacket) of the electron, we assume a Gaussian form 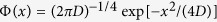, with 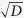 the width of the wavepacket. In weak coupling limit, which is properly characterized by 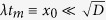 where *t*_*m*_ is the interaction time, short algebra yields^1^


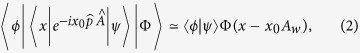


where 
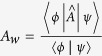
 is the AAV WV. We see that, indeed, the AAV WV manifests itself as a shift of the wavefunction. It seems that it is largely because of this feature that in ref. 10 the weak value shifts are emphasized as pre-existing. Or, the AAV WV is an effective coupling parameter to the meter system, e.g., via 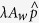
10. However, noting the ensemble-statistical interpretation of the quantum wavefunction, we find that this statement is not so different from the opinion by regarding the WV as the statistical average of measurement outcomes. Actually, if we measure the wavefunction in coordinate representation, the measurement outcomes satisfy the statistics with probability 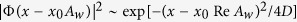. In the following, we will see that Re*A*_*w*_ is the lowest-order approximation of the conditional average of the measurement outcomes associated with the PPS statistics.

### Bayesian Treatment

To generalize the above analysis from weak coupling limit to finite strength interaction, the best way might be using the quantum Bayesian approach to calculate the PPS conditional average. In ref. 19 this kind of calculation was performed for a solid state qubit measured by quantum-point-contact^25^. Similarly, applying the quantum Bayesian rule for circuit-QED architecture^26^,^27^, the general expression of the associated weak values has been obtained^28^.

For the Stern-Gerlach setup, the transverse spatial coordinate of the electron plays the role of a meter which is further collapsed (measured) by an outside classical detector. The probability distribution of the measurement outcomes (the collapsed positions on the screen) is simply given by


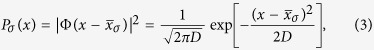


where 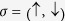, and 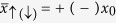 are the distribution centers associated with the states of spin-up 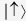 and spin-down 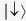.

If 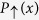 and 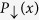 are strongly overlapped, the measurement (with outcomes of “*x*”) falls into the category of quantum *weak* measurement. In this case, the quantum Bayesian approach is also a perfect tool for the Stern-Gerlach setup. Originally proposed by Korotkov^25^, the quantum Bayesian approach is largely based on the well-known Bayes formula in Probability Theory together with a *quantum purity* consideration. The former is utilized to determine the diagonal elements while the latter is for determination of the off-diagonal ones. In quite compact form, one can use the quantum Bayesian rule to update the spin state from *ρ* to 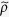 as follows:


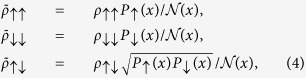


where 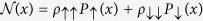 is a normalization factor. Note that the last equality (for the off-diagonal element) stems from the purity consideration^25^.

Precisely in parallel to the AAV’s treatment, let us consider the PPS states 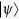 and 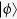, or in terms of the density matrices 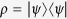 and 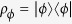. We will explicitly employ the PPS average of the measurement outcomes as the practical definition of weak values, which is actually in the same spirit of achieving the AAV WV and reads^19^,^20^,^21^,^28^


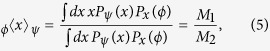


where 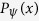 is the distribution probability of the measurement outcomes with the pre-selected state *ψ before the post-selection*. Note that, actually, 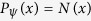. 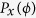 is the post-selection probability which can be obtained via 
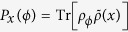
, by applying the quantum Bayesian rule Eq. (4), to update state from *ρ* to 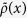 based on the outcome *x*. Obviously, 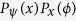 plays the role of the joint PPS probability of getting “*x*”, while having the denominator *M*_2_ as its normalization factor. Straightforwardly, by completing a couple of Gaussian integrals, the weak value defined by Eq. (5) is given by


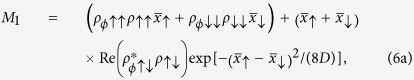



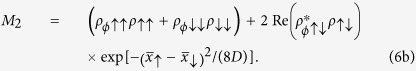


Where 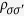 and 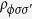 are the elements of the density matrices *ρ* and *ρ*_*ϕ*_, respectively.

To establish an explicit connection of the above generalized result with the AAV WV, we need to reexpress the result of Eq. (6). Without loss of generality, let us assume 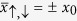. By expressing the AAV WV 
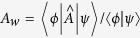
 in terms of the density matrix elements of *ρ* and *ρ*_*ϕ*_, after some simple algebra we obtain


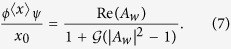


In this elegant result, we have introduced 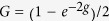 and 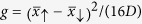.

We see that in the weak measurement limit (small *g*) and with modest 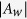 (not “strange” enough), Eq. (7) returns to the AAV’s result. This shows that the AAV’s WV (more precisely the real part of it) is indeed the PPS average of the measurement outcomes. Another important feature in the result of Eq. (7) is the second (correction) term in the denominator. It will make the PPS average considerably deviate from the AAV WV for finite strength measurement or with very “strange” 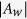. This feature should be kept in mind when one attempts to extract the AAV WV from the PPS average. Similar result as generalization of the AAV WV has been found as well for qubit measurements by quantum point contact^19^ and in the circuit-QED system^28^, and has been connected with the more general formulation of contextual values^20^,^21^.

In general, the AAV WV is a complex number. While Eq. (7) relates the PPS average with the real part of the AAV WV, how to relate it with the imaginary part of the AAV WV is of interest. In the context of optical (laser-beam) setup, it was shown in ref. 15 that one can “cleverly” post-select a specific transverse state corresponding to a specific position/momentum of the laser beam, in order to measure the real/imaginary part of the polarization WV. In ref. 29 the meaning and significance of the imaginary part of the AAV WV has been further exploited.

For the Stern-Gerlach setup, if one is able to introduce the “
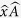
” -type interaction in the *system-meter* coupling Hamiltonian, i.e., 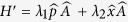, then the imaginary part of *A*_*w*_ can appear as well in the numerator of Eq. (7). To be specific, let us assume that the *system-meter*-coupling is switched on for a time interval *t*_*m*_. Then, *x*_0_ in the above 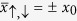 is given by 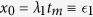. Moreover, when applying the quantum Bayesian rule Eq. (4), a phase factor 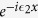 should be attached to 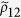 in the third equality, where 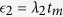. Inserting these accounts into the WV calculations, we obtain


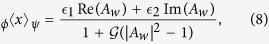


However, for the Stern-Gerlach setup, it seems unclear how to realize the above *dual* coupling Hamiltonian. Alternately, for the circuit-QED system as analyzed in ref. 28, it is indeed possible to obtain the WV expression as Eq. (8). There, even better, one can make either 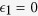 or 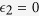 by tuning the local oscillator’s phase in the homodyne measurement of the cavity field.

### Origin of Anomalies

Following the standard and practical way of experimentally measuring the quantum average of a physical observable 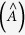, the above analysis established a general connection (for finite strength measurement) between the PPS average and the quantum AAV WV. From Eq. (7), we see that the extent of anomaly of the PPS average largely depends on *A*_*w*_. Actually, in the weak measurement limit, if we neglect the back-action (“disturbance”) effect of the measurement on the measured state, the rescaled PPS average 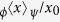 is precisely the AAV WV.

As a preliminary and most straightforward illustration, let us first inspect the origin of anomaly of the AAV WV which may be rewritten as


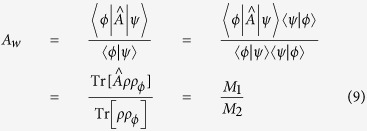


To be specific, consider the measurement of the real part of *A*_*w*_. We have 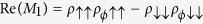 and 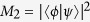. Let us also specify the PPS states as


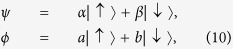


and assume that all the superposition coefficients (*α*, *β*) and (*a*, *b*) are real. Then we obtain 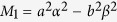 and 
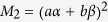
, and achieve the divergence condition 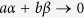, which corresponds to an ultra-small post-selection probability. In this case we also have 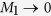 which, however, is a first-order small quantity while *M*_2_ is of the second order. This is essentially equivalent to the divergence feature of the AAV weak value *A*_*w*_, i.e., 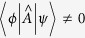 when 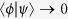.

Then, we see that it is right the *quantum interference* that possibly makes 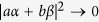, while 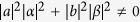. In classical case, the superposed *amplitudes* in the quantum states 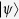 and 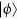 should be replaced by *probabilities* (modulus squares of the amplitudes). This would result in the joint PPS probability given by 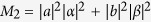. Therefore, we can definitely conclude that in classical case, the PPS average of 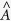 is *impossible* to exceed the normal bounds of 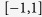, i.e., there is no *anomalous* classical weak values.

Now consider the finite strength measurement. Let us rewrite the joint PPS probability *M*_2_ in Eq. (6) as 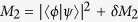, where


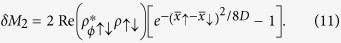


This “correction” (to the probability 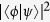) is originated from the “disturbance” (back-action) of the quantum measurement on the pre-selected state 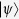, which alters the PPS probability by the amount of *δM*_2_. Notably, in a sharp contrast to the classical model to be discussed in the following, this “disturbance” is *not* at all the origin of causing anomalous weak values. Actually, owing to the presence of *δM*_2_, the PPS probability cannot approach zero. This implies that the WV is not to be divergent when 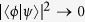. In contrast, noting that 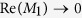 in this case, the presence of the nonzero *δM*_2_ will result in a vanishing WV^28^. The reason is that the “disturbance” can result in successful post-selection while it is impossible (owing to destructive quantum interference) if there is no disturbance. Finally we point out that, for finite strength measurement (in the presence of *δM*_2_, there still exists a window of post-selection and measurement strength for the appearance of *anomalous* WVs.

### Classical Coin-Toss Model

In the recent articles by Ferrier and Combes^7^,^8^, it was claimed that the anomalous weak values are not uniquely relate to quantum nature, but rather a purely statistical feature of pre- and post-selection with “disturbance”. Below we briefly revisit the classical *coin-toss* model analyzed in refs 7,8, in attempt to provide a simple view for the origin of the “anomaly” displayed there.

**Figure 2:**
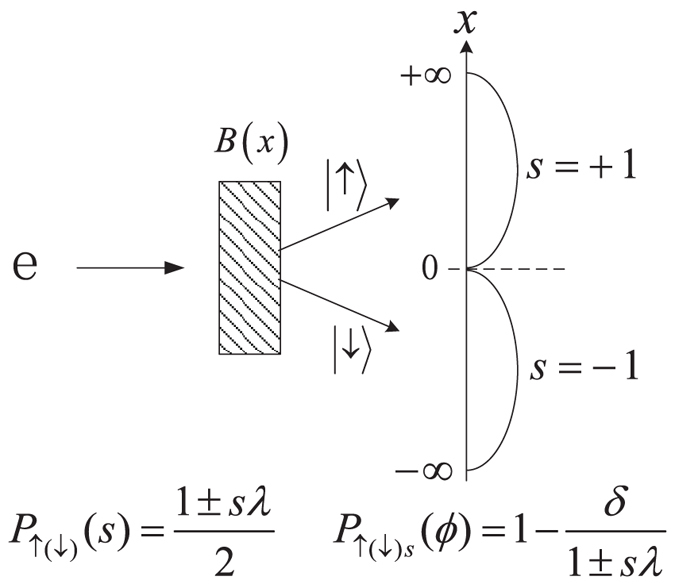
The coarse-grained version of the Stern-Gerlach setup which corresponds to the coin-toss model discussed in 7**, by regarding all the outcomes** 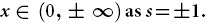 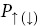
 is the “coarse-grained” spatial distribution probability of the “spin-up(down)” state, while 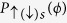 is its subsequent post-selection probability (note that it has an unusual/artificial feature of being *s*-dependent). *ϕ* is the post-selection state which was chosen as 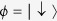 in ref. 7 (and in the present work), but in general which can be a classical mixture of 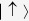 and 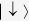.

The coin-toss model, which was originally analyzed in ref. 7, is actually a *coarse-grained* version of the Stern-Gerlach setup which we have discussed above. The probability of the coarse-grained outcome distribution of the “weak” measurements was proposed as^7^


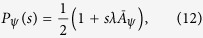


where 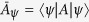 and *s* = ±1. Here, the coarse-grained variable “*s* = ±1” correspond to the integrated outcomes of 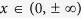. Note that in ref. 7, this coarse-grained variable was used in certain confusing manner together with the “Heads” and “Tails” in the coin-toss model, which hides then the serious artificial feature of post-selection rule assumed there. Below, to avoid such type of ambiguity, we use the terms of “spin-up” and “spin-down” for the “Heads” and “Tails”, i.e., the *intrinsic* coin states, while emphasizing *s* = ±1 for the meter’s coarse-grained outputs, as particularly shown by the plot of Fig. 2.

Now, more specifically, for the “spin-up” and “spin-down” coins, the coarse-grained locations obey the integrated probabilities in the two regions given by, respectively,


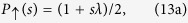



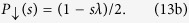


From these, one may clearly keep in mind that the “spin-up” and “spin-down” coins would locate in the “*s* = +1” and “*s* = −1” regions with different probabilities. One can easily check 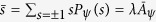. This implies that *λ* is the scaling parameter between the quantum expectation of 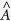 and the data average of meter’s outcomes. One can thus reasonably regard it as the measurement strength.

The key step in the WV analysis of the coin-toss model is to introduce a “disturbance” (bit-flip channel), which is modeled by


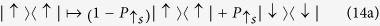



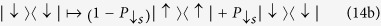


where


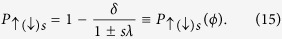


Note that in ref. 7 only the first process was explicitly displayed, because of 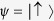 considered there. Obviously, *δ* characterizes the amount of disturbance and 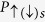 corresponds to the post-selection probability of 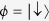 (hereafter we assume the post-selection state of 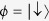, as the same in ref. 7).

To uncover the underlying problem more transparently, we would like to present a slightly generalized treatment by considering a superposition pre-selected state 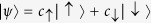, instead of 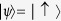 as in^7^. Now, applying the PPS average scheme


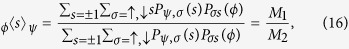


where 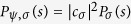, after simple algebra we obtain the same result of weak value as derived in ref. 7:


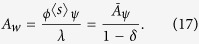


Remarkably, this classical WV can become “anomalous” and even be very “strange” by altering the parameter *δ*. This is the key result of ref. 7.

### Comparative Analysis

Based on the Bayesian treatment, we can convert the quantum result to its classical counterpart by simply dropping the off-diagonal terms in *M*_1_ and *M*_2_ in Eq. (6):


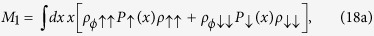



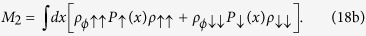


Accordingly, we obtain 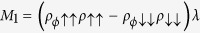 and 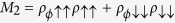. We see then that in classical case the PPS average cannot become *anomalous* since we always have 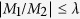.

Now let us reformulate the coin-toss model via


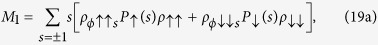



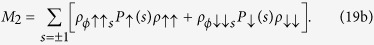


In the classical coin-toss analysis^7^, the most problematic procedure is the “insertion” of the following post-selection rule (the so-called “noisy channel” or “disturbance”):


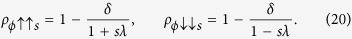


Accordingly, one gets 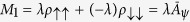 and 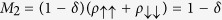. Then, by means of this procedure, one obtains anomalous WV since the scaled PPS average “
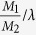
” can drastically exceed 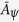.

Some remarks on the above post-selection “rule” are in order as follows. (i) Indeed, the overall post-selection probability 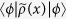 depends on the outcome “*x*” (or the coarse-grained location “*s*”). However, in either quantum or classical weak (“noisy”) measurement, this dependence has been fully accounted for by the Bayesian rule, via updating the state from *ρ* to 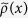. Then the post-selection probabilities from the component (basis) states 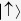 and 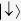, say, 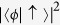 and 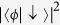, should no longer depend on “*x*” or “*s*”. In contrast, the post-selection probabilities given by Eq. (20) depend on *s* = +1 or *s* = −1. This is a misleading procedure to “generate” the anomalous classical WV. The “rule” of Eq. (20), or any other “*s*-dependent rule”, is an artifact, which simply means keeping or discarding the stochastic events (*s* = ±1) according to our willing, then re-calculating the average of *s* and extracting the weak values as 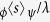. Obviously, it does not make sense by comparing this type of PPS average with the quantum WV. (ii) So we should obey the *convention* that the post-selection probabilities 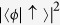 and 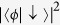 do *not* depend on “*x*” or “*s*”. Under this requirement, as proved below Eq. (18), we conclude that it is *impossible* to generate anomalous WV in any classical contexts. (iii) In order to get anomalous WV, the only way is adding the interference terms into *M*_2_ [c.f. Eqs. (6) and (18)], to make 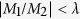. This is possible only in quantum case. So the anomalous WV is indeed a *unique* quantum phenomenon, which deeply originates from quantum interference, or the most fundamental quantum superposition principle. Actually, it was proved in ref. 19 that the anomalous WV is equivalent to the violation of the Leggett-Garg inequality, which is also a direct consequence of the quantum superposition principle. (iv) The anomalous WV is owing to the distortion of the joint PPS probability distribution, which is caused by quantum interference in quantum system but in the classical coin-toss model by an artificial procedure. In ref. 7 the “post-selection” was termed as “disturbance” (or “noisy channel”). However, for the problem under consideration, the only acceptable “disturbance” is the measurement (or information-gain) backaction, which has been fully accounted for by the Bayesian rule, for both the quantum and classical measurements. As clearly seen in the quantum WV analysis in this work, this type of “disturbance” would reduce the “anomaly” amount, which is in sharp contrast with the coin-toss model where the extra “disturbance” is the key reason of generating the “anomaly”.

## Discussion

To summarize, we have presented a simple and direct method to revisit the concept of quantum weak values. The Bayesian treatment associated analysis in comparison with a controversial classical model supports the assertion that the anomalous weak values are purely quantum mechanical, having no classical analogue. That is, in addition to the argument of *functional* dependence^10^,^13^, we arrive to a stronger conclusion: the anomalous WVs cannot be reproduced by any *correctly* treated classical model.

This conclusion is in full agreement with the finding uncovered in ref. 19, where the equivalence proof between the anomalous WV and the violation of Leggett-Garg inequality implies that the anomalous WVs rule out any classical (hidden-variable) interpretation. Therefore, an insistence that disallows adding extra disturbance is to make the classical-quantum comparison at equal foot. The reason is just like the following: if one introduces “extra procedures” in the (classical) hidden-variable treatment, it would destroy the meaning of violation of the Bell-Leggett-Garg inequalities.

In quantum mechanics, the wave function is actually a “knowledge” which is to be altered after measurement. This is the so-called backaction or disturbance of quantum measurement. For the *noisy* measurement in the classical coin-toss model, the measurement outcome will also change the prior probability (“knowledge”) known before the measurement. So in this sense a noisy classical measurement resembles the quantum measurement — both obey the Bayesian rule. This explains further that in any classical model, a correct treatment should disallow adding extra “disturbance”, since the classical “information-gain backaction”, which corresponds to the quantum measurement backaction, has been accounted for by the Bayesian rule, as clearly analyzed in our work by Eqs. (18) and (19).

## Additional Information

**How to cite this article**: Qin, L. *et al.* Simple understanding of quantum weak values. *Sci. Rep.* **6**, 20286; doi: 10.1038/srep20286 (2016).

## References

[b1] Aharonov Y., Albert D. Z. & Vaidman L. How the result of a measurement of a component of the spin of a spin-1/2 particle can turn out to be 100. Phys. Rev. Lett. 60, 1351 (1988). 10038016 10.1103/PhysRevLett.60.1351

[b2] Aharonov Y. & Vaidman L. Properties of a quantum system during the time interval between two measurements. Phys. Rev. A 41, 11 (1990). 9902834 10.1103/physreva.41.11

[b3] Duck I. M., Stevenson P. M. & Sudarshan E. C. G. The sense in which a “weak measurement” of a spin-1/2 particle’s spin component yields a value 100. Phys. Rev. D 40, 2112 (1989). 10.1103/physrevd.40.2112 10012041

[b4] Leggett A. J. Comment on “How the result of a measurement of a component of the spin of a spin-1/2 particle can turn out to be 100”. Phys. Rev. Lett. 62, 2325 (1989). 10039915 10.1103/PhysRevLett.62.2325

[b5] Aharonov Y. & Vaidman L. Aharonov and Vaidman reply. Phys. Rev. Lett. 62, 2327 (1989). 10039917 10.1103/PhysRevLett.62.2327

[b6] Aharonov Y. & Vaidman L. The two-state vector formalism: an updated review. arXiv:0105101; Lect. Notes Phys. 734, 399–447 (2008).

[b7] Ferrie C. & Combes J. How the result of a single coin toss can turn out to be 100 heads. Phys. Rev. Lett. 113, 120404 (2014). 25279610 10.1103/PhysRevLett.113.120404

[b8] Ferrie C. & Combes J. Classical correlation alone supplies the anomaly to weak values. arXiv:1410.8067v1.

[b9] Hofmann H. F., Iinuma M. & Shikano Y. Why the “classical” explanation of weak values by Ferrie and Combes does not work: a comment on Phys. Rev. Lett. 113, 120404 (2014). arXiv:1410.7126v2.

[b10] Vaidman L. Comment on “How the result of a single coin toss can turn out to be 100 heads”. arXiv:1409.5386v1. 10.1103/PhysRevLett.114.118901 25839317

[b11] Aharonov Y. & Rohrlich D. Comment on “How the result of a single coin toss can turn out to be 100 heads”. arXiv:1410.0381.

[b12] Brodutch A. Comment on “How the result of a single coin toss can turn out to be 100 heads”. Phys. Rev. Lett. 114, 118901 (2015). 25839317 10.1103/PhysRevLett.114.118901

[b13] Dressel J. Weak values as interference phenomena. Phys. Rev. A 91, 032116 (2015).

[b14] Kofman A. G., Ashhab S. & Nori F. Nonperturbative theory of weak pre- and post-selected measurements. Phys. Rep. 520, 43 (2012).

[b15] Dressel J., Malik M., Miatto F. M., Jordan A. N. & Boyd R. W. Colloquium: Understanding quantum weak values: basics and applications. Rev. Mod. Phys. 86, 307 (2014).

[b16] Kirkpatrick K. A. Classical three-box ‘paradox’. J. Phys. A: Math. Gen. 36, 4891900 (2003).

[b17] Dressel J. & Jordan A. N. Sufficient conditions for uniqueness of the weak value. J. Phys. A: Math. Theor. 45, 015304 (2012).

[b18] Ravon T. & Vaidman L. The three-box paradox revisited. J. Phys. A: Math. Theor. 40, 2873 (2007).

[b19] Williams N. S. & Jordan A. N. Weak values and the Leggett-Garg inequality in solid-state qubits. Phys. Rev. Lett. 100, 026804 (2008). 18232905 10.1103/PhysRevLett.100.026804

[b20] Dressel J., Agarwal S. & Jordan A. N. Contextual values of observables in quantum measurements. Phys. Rev. Lett. 104, 240401 (2010). 20867281 10.1103/PhysRevLett.104.240401

[b21] Dressel J. & Jordan A. N. Contextual-value approach to the generalized measurement of observables. Phys. Rev. A 85, 022123 (2012).

[b22] Pusey M. F. Anomalous weak values are proofs of contextuality. Phys. Rev. Lett. 113, 200401 (2014). 25432026 10.1103/PhysRevLett.113.200401

[b23] Romito A., Jordan A. N., Aharonov Y. & Gefen Y. Weak values are quantum: you can bet on it. arXiv:1508.06304.

[b24] Aharonov Y., Popescu S. & Tollaksen J. A time-symmetric formulation of quantum mechanics. Physics Today 63, 27 (2010). 10.1103/physreva.52.2538 9912531

[b25] Korotkov A. N. Continuous quantum measurement of a double dot. Phys. Rev. B 60, 5737 (1999).

[b26] Korotkov A. N. Quantum Bayesian approach to circuit QED measurement. arXiv:1111.4016.

[b27] Wang P., Qin L. & Li X. Q. Quantum Bayesian rule for weak measurements of qubits in superconducting circuit QED. New J. Phys. 16, 123047 (2014), Corrigendum: Quantum Bayesian rule for weak measurements of qubits in superconducting circuit QED. *ibid.* **17**, 059501 (2015).

[b28] Qin L., Liang P. & Li X. Q. Weak values in continuous weak measurement of qubits. Phys. Rev. A 92, 012119 (2015).

[b29] Dressel J. & Jordan A. N. Significance of the imaginary part of the weak value. Phys. Rev. A 85, 012107 (2012).

